# The Wrong Turning in Public Health

**Published:** 1922-10

**Authors:** Fred. E. Wynne

**Affiliations:** (Medical Officer of Health for Sheffield, and Professor of Public Health, University of Sheffield)


### THE WRONG TURNING IN PUBLIC HEALTH.

**By FRED. E. WYNNE, M.A., B.Ch., D.P.H. (Medical Officer of Health for Sheffield, and Professor of Public Health, University of Sheffield).**

THE administration of public health in its beginnings, and indeed up to quite recent times, was essentially what it was often called—"preventive medicine." That term has almost dropped out of use, and the "wrong turning" is the tendency for the ideals of prevention to be obscured in our solicitude for the treatment of individuals already diseased.

Communal disease is the last term in a faulty social metabolism; to alleviate or cure it in the individual is the function of the physician and the surgeon. To seek its origin, to discover where the social metabolism goes wrong, to direct our social evolution along the channels that make for health in the community, is the function of government, both national and municipal.

The principal cause of communal disease is poverty, and poverty is increased by waste. Excessive taxation is one of the causes that is preventing trade recovery, and is thus both directly and indirectly increasing and maintaining poverty; and with poverty, public ill-health. Every penny spent on public health administration, as on education or any other object admirable in itself, is waste, if it cannot be justified by results. When Government or municipal organisations begin to deal with individuals, the tendency to waste is enormously multiplied, for public funds can never deal with the whole of the individuals suffering from disease. We shall always be attacking our problems on mere fragments of the periphery, and, as can already be seen the cost of this kind of skirmishing is so excessive that in these days funds must be diverted from what should be the main attack on disease at its source and origin. We have thus in the promotion of public health a greater backing of enthusiasm than probably our predecessors ever knew. But enthusiasm to be effective must be guided by expert criticism, and I think it is the duty of public health administrators to resist the temptation to yield to popular clamour in favour of purely eleemosynary schemes, or methods of advertisement, which tend to divert public funds from the prevention of disease to tinkering with disease when it has developed.

The principal developments in public health administration of late years have been the National Insurance Act, with its provisions for the treatment of sickness generally, and in especial of tuberculosis; the medical inspection and treatment of school children; the great development of maternity and child welfare work; and, lastly, the free treatment of venereal diseases. The whole of this work, all most laudable in intention, is directed to the individual. Its cost has been almost incalculable, yet when it was found necessary to tackle the impossible environmental conditions into which our population had drifted at the termination of the war, the whole of our housing schemes broke down for want of money. Overcrowding and slum conditions are to-day engendering tuberculosis, drunkenness, venereal diseases, rickets, pneumonia, measles, and other diseased conditions in quantities which our breathless efforts at dealing with end-results are powerless to overtake.

On the other hand, public health administration of the old-fashioned sort aimed almost entirely at the environment. The improvements effected by it affected whole masses of the population, not selected individuals.

Public health in the sense in which we know it, including all factory and industrial legislation, is practically the product of the nineteenth century. An unexampled increase of population resulted from the industrial revolution, and an utter want of foresight permitted this population to develop uncontrolled in conditions of squalor almost inconceivable. The resulting epidemics of fever and cholera terrified our rulers into legislation directed to the improvement of the environment of the people. The various measures passed during the first three-quarters of the century were consolidated in the Public Health Act of 1875, which has from time to time been amended, and was quickly followed by further legislation in the Infectious Diseases Prevention Act and the Housing Acts.

Every medical officer of health knows the depressing effect, the sense of almost hopelessness, that is apt to crush him in his visits to insanitary property, the indignation that is apt to possess the twentieth-century bosom in the presence of overcrowding, dilapidation, dampness, darkness, dirt, unpaved yards, privy-middens, open ash-pits, above all the acquiescence in squalor, the indifference to filth, the utter carelessness that makes the worst of foul conditions which is displayed by a generation that is the product of over fifty years of what we retain the habit of describing as "education."

No one who is familiar with the Public Health Act, or who has studied the conditions with which early public health legislation had to cope, can fail to be impressed with the comprehensive and radical ideas of the men who framed it. In all the legislation of that period, almost the only attempt to deal with the individual was the provision of isolation hospitals for infectious disease, and that was undertaken primarily not in the interest of the patient, but for the protection of the community against the spread of the disease. Even vaccination, though necessarily applied to the individual, was, in the days before sanity was ousted by sentimentality, made compulsory for the whole population. It is interesting, therefore, to glance at the results achieved by this tremendous attack on environment and to compare them with the results so far achieved by our modern method of treating the individual.

The history of the general death-rate in England and Wales during the fifty-two years 1870 to 1921 shows that the highest figure was 23, in 1870. This is almost equalled in 1875, the date of the passing of the Public Health Act, and from that time on the decline has been continuous, so that in 1921 the rate is practically only one-half that of 1870—namely, 12 per 1,000, against 23 per 1,000.

The history of the decline in the death-rate from some of the more important infectious diseases from 1851 to 1920—a period of seventy years—shows that at all ages the death-rate from the group of diseases known as "Fever" has declined from 980 to 25 per million. Small-pox, with a sudden rise in the late sixties, has declined from 250 per million to nothing; enteric from 375 in 1870 to 25, and typhus from 75 in 1870 to nothing In the age groups 0 to 15, the mortality from scarlet fever has declined from 2,700 in 1865 to 25 in 1919. This is without any corresponding declension in the attack rate: it is almost entirely a lessened fatality representing a change in the type of the epidemics. The only factors we know of to account for this are environmental improvements and isolation in hospital. It seems not unlikely that as the more severe cases would be more certainly diagnosed and isolated, it was the milder strains, the missed cases, that had the opportunity of propagating a milder type of the disease. Diphtheria has declined from 1,400 in 1865 to 500. Here, of course, an important factor has been the introduction of anti-toxin, essentially an example of treatment of the individual, but the high death rate from this cause remains an unsatisfactory feature of our statistics. Measles, for which nothing has been done directly, has hardly declined at all. The comparatively low figure of 600 in 1919 has no guarantee of permanency, and measles remains the most deadly disease of infancy; all the more dangerous because it seems impossible to teach the public that it is not a trifling disease, although even in the sixties it was claiming nearly five times as many victims as small-pox.

These are records of great achievement, and it would, of course, be unfair to expect the curve of progress to maintain the same steep angle. It is also impossible to make a just comparison between recent developments, with a record of only a few years, and the comparatively long periods we have been discussing.

In the decline in infant mortality from 1870 to the present day, what I may call the individualist school of public health has its greatest triumph. Investigation shows that all the sanitary work of the last thirty years of the nineteenth century did nothing at all to protect or prolong infant life ; in fact, during this period it actually reached its maximum in 1899.

This is not very suprising, for the child's environment is its mother or its nurse, and to this day hygiene and mother-craft are not considered "educational subjects." The management of infants remained a matter of tradition expounded by the handy woman and her colleague "the lady next door." Doctors who seldom saw a sick baby before they were qualified absorbed their clinical teaching from the same exalted source. Then, of course, the child had to swallow drugs of one sort or another, or wherewithal could the quarterly bill be furnished forth ? Then when the child was weaned, prematurely or otherwise, the only available food was the emulsion of cow-dung and house-flies known as fresh milk.

Our child welfare centres have done an immense amount to change this state of affairs. To them and their staffs, and to the unremitting toil and devotion of armies of health visitors, must, I think, be given the main credit for the fact that the infantile mortality of England and Wales in 1921 was almost precisely half what it was in 1899. The very fact that we are causing so many infants to survive seems to me only to emphasise the duty of providing them with an environment in which they can be happy.

The story of the death-rate from pulmonary and other forms of tuberculosis from 1859 to the present shows very clearly the seldom appreciated fact that tuberculosis was a steadily decreasing disease for at least a generation before the public became excited about it. The whole of our tuberculosis administration is a classic example of the ill effects of neglecting environment in favour of dealing with the individual. Well-intentioned but untrained people awoke to the fact that very large numbers were dying of tuberculosis, and about the same time open-air treatment and sanatoriums were boomed as a method of cure. There was an outbreak of "national societies" through which influential members of society demanded the immediate provision of sanatoriums for all, and this to a great extent was done at an enormous cost in the days when there was "money to burn." The thing must be done on a "national" scale ; there was to be no waiting for the result of experiment on a smaller scale, and the still small voice of criticism and warning was ignored. But the warning voice was there. Many who had long experience of tuberculosis were sceptical, and as long ago as 1910, when the Insurance Bill was still under discussion, Messrs. Elderton & Paten had published the third series of their "Study of the Statistics of the Mortality of the Tuberculosis and Sanatorium Treatment," under the auspices of the Department of Applied Mathematics of London University. Their conclusions were as follows :—

1. The mortality of tuberculous patients who are undergoing or have undergone treatment is much heavier than that of the general population, and even when the disease is taken in an incipient stage the mortality is about four times as heavy.

2. The mortality of the apparently cured cases is about twice as heavy as that of the general population.

3. The mortality among sanatorium patients does not show any improvement on that of Williams and Pollock's cases—that is, cases treated in pre-sanatorium days.

These conclusions are fully borne out by the more recent statistics of Dr. Bardswell and others, and I think it is now commonly held that the most hopeful function of the sanatorium is the isolation of infective cases. Yet we have been landed in the position of spending some millions a year on the treatment of tuberculosis, while there is no money available for the elimination of the insanitary areas where we cultivate more tuberculosis in a week than all the sanatoriums could house in a year.

I must now refer briefly to that very important branch of modern public health work—the school medical service. Here again I speak from an experience of over ten years as a school medical officer, and I am satisfied that as a result of the work of the school medical service a very large number of individuals has been benefited. We have only to read through Sir George Newman's most valuable reports to realise the vast scale on which the work is now organised; a development of which some measure may be found in the fact that the cost of the service has increased from £325,735 in 1913 to £885,625 in 1920.

Even the latter figure would be by no means excessive if it were producing a harvest of health, if it were being spent in directions which could be relied on to produce successive crops of school children with a declining sickness rate, so that the cost of discovering and treating ailments would be a steadily diminishing amount. But among all the tables published in these annual reports I can find none that shows a comparison between the percentage of children found to be suffering from some form of defect in each of the years since school medical inspection has been in force. The materials for such a table must be available, but there is no reason to suppose that it would show any decline. The number of cases of disease treated in one generation of school children will not lessen the number of defects in the next.

The best test of the educational success of the school medical service is probably the promotion of personal cleanliness and the reduction of verminous conditions. The diagnosis of vermin is still met by parents as an insult, and denied even in the face of the demonstration of lice actively parading the scalp. Sir George Newman, in his 1917 Report, was evidently deeply impressed with the importance of environment. He says :—

"Here is an unclean child to be cleansed by the authority, but it returns to a verminous home.

Here is a child provided with spectacles, but it continues to be taught to read small type in a badly lighted classroom. Here is a scheme for providing school meals, but the arrangement bears no relation to the food supply of the home of the child or the social condition in which the child lives."

Again he says :—

"It is well to cure ringworm, remove adenoids, and correct defective vision; but it is still more important by unity of effort and co-ordination of agency to secure the essentials of health for the whole body of the six million children in the State schools."

Yet in 1920 he tells us, "The school medical service finds its fulfilment only in the service it can render to the individual child."

It is only a few months ago that I had to protest in vain against the continued use of a school where the only playground for boys was an underground space between the foundations of the building. On a bright summer day there was only the light available from some narrow dirt-encrusted windows on the level of the adjoining footpath, and into this space both urinals and water-closets ventilated direct. I was told that it was impossible to close the school because there was no other accommodation for the children, and as to making improvements—it was a "non-provided" or denominational school, and the managers had no funds. That, of course, is typical of hundreds of schools all over the country. If the millions that have been spent on medical inspection and treatment had been spent on knocking these places down and building decent schools, schools comparable to the modern admirable "Council schools," we should then have had some right to start curing ailments as they arose. As it is we compel thousands of our children to spend a quarter of their childhood under conditions of sanitation which if permitted in a prison would set every sentimentalist in the country howling with indignation.

There is no reference in the annual reports of the Chief Medical Officer to the sanitary conditions of existing schools, for the simple reason that no reference to this painful subject is made in the individual reports of the school medical officers; and I know why. I once published an accurate survey of the sanitary conditions of the schools in my area. I was young then and did not understand the mentality of our "educationists." But I never did it again, and would advise my colleagues to postpone any such report until they are on the eve of resigning their appointment. Perhaps the National Union of Teachers is strong enough to take the matter up in the interests of its own members as well as of their pupils. Personally I hold that the time has gone by when children should be forced into utterly insanitary surroundings in the interest either of sectarian religion or the process described as education, and to give "instruction in hygiene" under such circumstances is merely a cynical farce. It is the old story of deliberately creating disease and establishing costly methods of treating it. If the treatment is carried out by a State service it is "preventive"; presumably if done by the general practitioners it does not deserve this label.

Time forbids anything but a passing reference to our latest and probably our least successful "stunt"—the campaign against venereal disease, on which we are now spending, I believe, a little short of a million a year, but it is difficult to obtain anything like accurate figures. Now that venereal disease has become a fashionable topic for drawing-room conversation, it is surely time that we pulled our heads out of the sand, adopted compulsory notification, and made the subjects of disease pay for their treatment according to their means. Here again we preach a conventional morality to people for whom we have made marriage a practical impossibility by the promotion of poverty and the failure to provide economic housing.

My conclusion from all these considerations is that we have turned away from the ideals of the Public Health Act, from the improvement of environment which produced such incontestably good results, long before work in this direction was completed—when, in fact, it was little more than begun—and that we are depleting the funds required for this work by a costly and partial treatment of the end-results of the processes inaugurated by faulty environment; that we are bleeding the taxpayer and the ratepayer by expenditure that does not give an adequate return in improved health and prolonged life; and that we are undermining responsibility by providing services at the public expense which should be paid for by the individual.

